# A simple and dual expression plasmid system in prokaryotic (*E*. *coli*) and mammalian cells

**DOI:** 10.1371/journal.pone.0216169

**Published:** 2019-05-02

**Authors:** Manabu Murakami, Takayoshi Ohba, Agnieszka M. Murakami, Chong Han, Kenji Kuwasako, Shirou Itagaki

**Affiliations:** 1 Department of Pharmacology, Hirosaki University Graduate School of Medicine, Hirosaki, Japan; 2 Department of Cell Physiology, Akita University Graduate School of Medicine, Akita, Japan; 3 Frontier Science Research Center, Department of Pathology, Faculty of Medicine, University of Miyazaki, Miyazaki, Japan; 4 Collaboration Center for Community and Industry, Sapporo Medical University, Sapporo, Japan; IRIG-CEA Grenoble, FRANCE

## Abstract

We introduce a simple and universal cloning plasmid system for gene expression in prokaryotic (*Escherichia coli*) and mammalian cells. This novel system has two expression modes: the (subcloning) prokaryotic and mammalian modes. This system streamlines the process of producing mammalian gene expression plasmids with desired genes. The plasmid (prokaryotic mode) has an efficient selection system for DNA insertion, multiple component genes with rare restriction sites at both ends (termed “units”), and a simple transformation to mammalian expression mode utilizing rare restriction enzymes and re-ligation (deletion step). The new plasmid contains the lac promoter and operator followed by a blunt-end EcoRV recognition site, and a DNA topoisomerase II toxin-originated gene for effective selection with isopropyl-β-D-thiogalactoside (IPTG) induction. This system is highly efficient for the subcloning of blunt-end fragments, including PCR products. After the insertion of the desired gene, protein encoded by the desired gene can be detected in *E*. *coli* with IPTG induction. Then, the lac promoter and operator are readily deleted with 8-nucleotide rare-cutter blunt-end enzymes (deletion step). Following re-ligation and transformation, the plasmid is ready for mammalian expression analysis (mammalian mode). This idea (conversion from prokaryotic to mammalian mode) can be widely adapted. The pgMAX system overwhelmingly simplifies prokaryotic and mammalian gene expression analyses.

## Introduction

Numerous commercial expression plasmids exist, especially for mammalian transient expression. The process of mammalian transient expression of a desired gene has mainly relied on a two-step method: subcloning of the desired gene into a subcloning plasmid such as pBluescript (Agilent Technologies, Santa Clara, CA, USA), and subcloning the desired gene into a mammalian expression plasmid such as pcDNA3 (Thermo Fisher Scientific, Waltham, MA, USA) [1]. Each cloning step is often troublesome, due to the low efficiency of DNA ligation. There are several methods for the first subcloning step, including the traditional blunt-end treatment of a polymerase chain reaction (PCR) product with T4 DNA polymerase, ligating a fragment into a blunt-end EcoRV site in a pBluescript plasmid, or restriction enzyme digestion and ligation. Developments in molecular biology have enabled other cloning methods, such as TA, TOPO and Gateway cloning, although these have relatively high costs and are dependent on PCR [2–5]. TA cloning uses Taq DNA polymerase to add a single adenosine-residue overhang to the 3' end of the PCR product. As Taq DNA polymerase lacks 3' to 5' exonuclease proofreading activity, it has relatively low fidelity. Furthermore, a single adenosine overhang becomes degraded over time, which reduces the ligation efficiency. TOPO cloning uses topoisomerase to unwind and ligate the DNA. Gateway cloning has the benefit of a single recombination reaction that moves a section of DNA from one plasmid to another. For rapid construction of multiple DNA fragment ligations, both Gibson Assembly and Golden Gate Assembly are available, although they require specific enzymes [6, 7]. Another ligation method for multiple DNA fragments, the SLIC method, has also been established [8]. This method is cost effective and dependent on PCR and T4 exonuclease activity.

Nevertheless, it is usually necessary to first transfer the desired gene into a subcloning plasmid. After successful subcloning, the desired gene is treated by restriction enzyme digestion, agarose gel electrophoresis, DNA purification from an agarose gel fragment, and ligation into an expression vector. Otherwise, the subcloned genes can be used for further PCR-based plasmid construction. Here, we report a new type of plasmid, pgMAX, which enables highly efficient subcloning, expression of the desired gene with IPTG induction in *Escherichia coli*, and the simple and rapid construction of mammalian expression plasmids.

## Methods

### Plasmid construction

Bicistronic expression vectors, pIRESpuro3 and pZero-2, were purchased from Thermo Fisher Scientific. DsRed2 and pEGFP-N1 plasmids were purchased from Clontech Laboratories (Palo Alto, CA, USA) [9]. The Bgeo gene (a combined gene of β-galactosidase and neomycin phosphotransferase) was kindly provided by Dr. Smith [10]. For plasmid construction, PCR-based mutagenesis was performed. The conditions for PCR with high-fidelity Pfu DNA polymerase (Agilent Technologies) were empirically modified (denaturation at 94°C for 20 s, an annealing step at the calculated temperature (ca. 50°C) for 30 s and an extension at 72°C for 30 s, for 35 cycles). Amplified PCR products were gel-purified with a gel extraction kit (Macherey-Nagel GmbH, Dueren, Germany). The inhibitory unit (iUnit) was PCR-amplified from pZero2 (Invitrogen, Carlsbad, CA, USA)) with a specific oligo DNA (iFor and iRev, see S1 Table). Additional restriction enzyme sites were introduced with oligonucleotide DNA primers (S1 Table), and protocols using the pgMAX system as well as the entire pgMAX sequence have been deposited in protocols.io. (DOI dx.doi.org/10.17504/protocols.io.zq3f5yn). The SwaI restriction site; lac promoter (lacP) and operator (lacO); Kozak sequence (Kozak), PmeI restriction site; BamHI site; Flag protein tag; BamHI site; and EcoRI, EcoRV and XhoI restriction sites were constructed using a PCR. The blunt-end product was inserted into the EcoRV site in pBluescriptIISK^-^ (Agilent Technologies) using standard ligation techniques (Takara DNA Ligation kit ver.2.1, Takara, Otsu, Japan). For the transformation, XL10-Gold ultracompetent cells (Tetr Δ(mcrA)183 Δ(mcrCB‒hsdSMR‒mrr)173 endA1 supE44 thi‒1 recA1 gyrA96 relA1 lac Hte [F'proAB lacIqZΔM15 Tn10 (Tetr) Amy Camr] (Agilent Technologies) were used.

A blunt-end DsRed2 DNA fragment was amplified using Pfu DNA polymerase with DsRed2-specific oligo DNA (DsRed2for: AaaGCTAGCatgGCCTC CTCCGAGAAC GTCATCA; DsRed2rev: aaaGAATTCagatctcaggaacaggtggtg). A blunt-end enhanced green fluorescent protein (EGFP) DNA fragment was amplified using high-fidelity Pfu with EGFP-specific oligo DNA (EGFPfor: cccGCTAGCatgGTGAGCAAGGGCGAGGAG; EGFPrev: cccGGTACCGGCGGCGGTCACGAACTCCAG). The PCR-amplified product was inserted into the EcoRV site of pgMAX.

### Cell culture and transfection

Cell culture and lipofection were performed as described previously [11]. Human embryonic kidney cells (HEK293, ATCC CRL 1573) were cultured in Dulbecco’s Modified Eagle’s Medium supplemented with 10% fetal bovine serum. Exponentially growing cells were plated onto 35-mm dishes, and lipofection was performed using commercially prepared lipofectamine (Invitrogen).

### Microscopy

Standard epifluorescence optics (Olympus, Tokyo, Japan) was used to visualize DsRed2 (excitation wavelength 563 nm, emission wavelength 582 nm) or EGFP (excitation wavelength 470 nm, emission wavelength 505 nm). DsRed2-related fluorescence was recorded with a Hamamatsu ORCA-FLASH 4.0 system (Hamamastu Photonics, Hamamatsu, Japan).

### RNA isolation and reverse transcription (RT-PCR) analysis

Total RNA was isolated from the HEK 293T cells using an RNeasy extraction kit (Qiagen Inc., Valencia, CA, USA). The reverse transcription reaction was performed in a solution of 10 pmol oligo-dT primer, 1 μg RNA, 1 x first strand cDNA buffer (Life Technologies, Rockville, MD, USA), 10 mM dithiothreitol, 0.4 mM dNTPs and 200 U Superscript III (Thermo Fisher Scientific), in a volume of 20 μl, at 42°C for 45 min. DsRed2 -specific sequences were amplified by PCR (30 cycles) with DsRed2for andDsRed2rev primers, which generated a PCR product of 699 bp for the comparative RT-PCR analysis.

### Western blot analysis

Cell lysates from cell lines were prepared using TNE buffer in the presence of protease inhibitor cocktails (*Roche* Pharma, Basel, Switzerland). Lysates (100 μg) were subjected to sodium dodecyl sulfate-polyacrylamide gel electrophoresis (SDS-PAGE, 15%) and blotted with the following antibodies: anti-Flag (Sigma-Aldrich, St. Louis, MO, USA), anti-DsRed (Arigo Biolaboratories Corp., Hsinchu City, Taiwan) and anti-GAPDH (Abcam Inc., Cambridge, UK) and were then incubated with alkaline phosphatase-conjugated secondary antibodies. The results were visualized by a colorimetric reaction using Western Blue Stabilized Substrate for Alkaline Phosphatase (Promega, Madison, WI, USA).

For Western blot analysis using *E*. *coli*, 0.5 ml of the culture medium (LB medium at 37°C, 12–16 h) was harvested and resuspended in 100 μl lysis buffer (1 mM EDTA, 1 mg/ml lysozyme) and incubated at room temperature for 15 min. Aliquots (10 μl) of the homogenate from each clone were resolved by 15% SDS-PAGE and subjected to Western blotting. A commercially available polyclonal antibody specific for the Ca^2+^ channel β_2_ subunit (Sigma-Aldrich) was used, followed by a secondary anti-rabbit IgG antibody conjugated to alkaline-phosphatase (Promega, Madison, WI, USA).

### Statistics

Data are expressed as the means ± the standard error of the mean. Prior to statistical analyses, data were analyzed with the Shapiro-Wilk test. After confirmation of a normal distribution, statistical differences were further determined by Student’s *t*-test or an analysis of variance with a Bonferroni post hoc test. *P* < 0.05 was considered to indicate statistical significance.

## Results

### Plasmid construction

**Fig 1:**
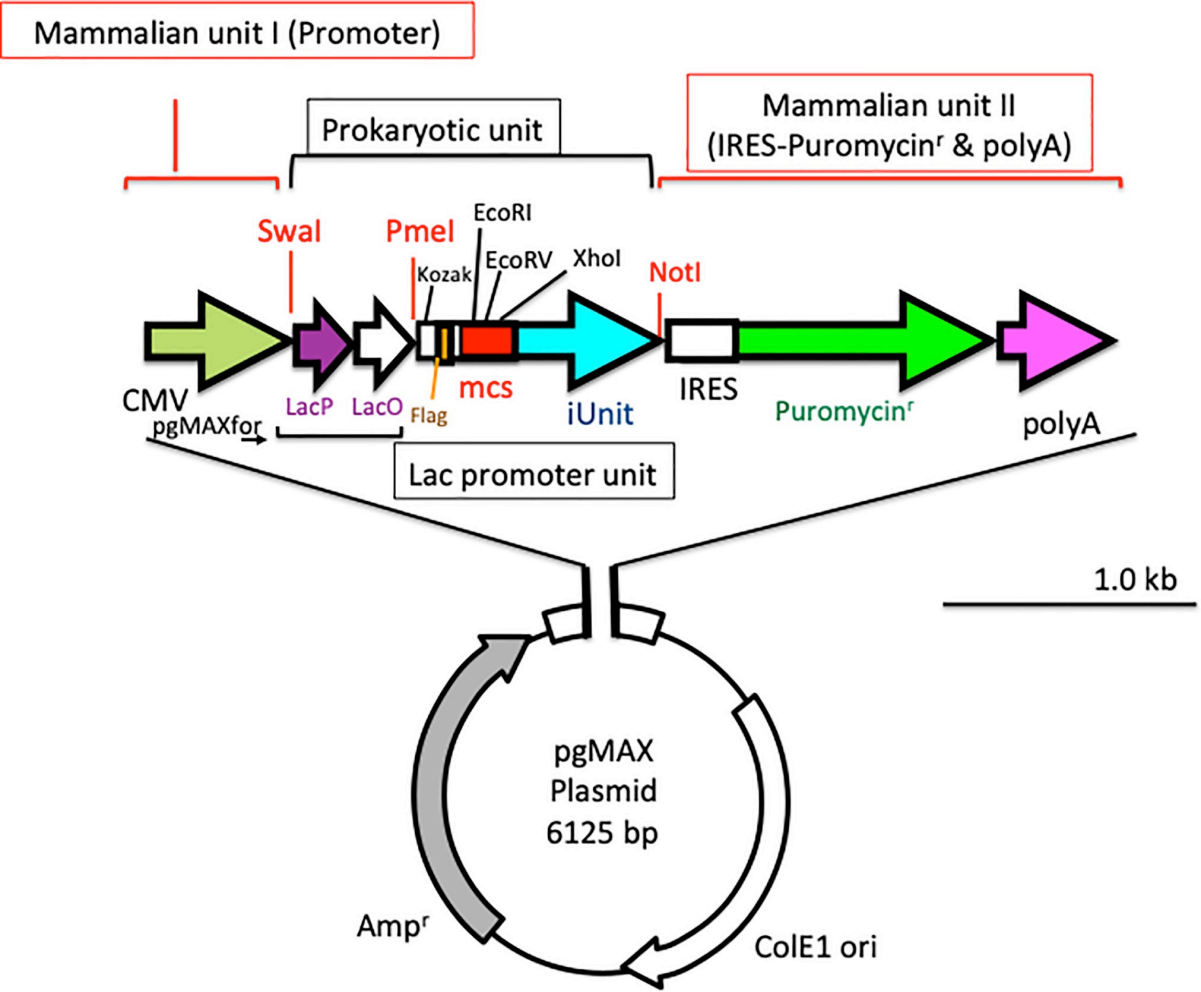
The pgMAX plasmid in prokaryotic expression mode. The pgMAX promotor has two functional components, prokaryotic and mammalian expression. The promoter is composed of a CMV promoter and IRES-puromycin N-acetyl-transferase gene (green arrow) with a poly A tail (pink arrow)(mammalian units I and II). The restriction enzyme (SwaI, PmeI, EcoRI, EcoRV, XhoI and NotI) sites are indicated. Oligo DNA for PCR screening is also indicated (pgMAXfor).

Fig 1 shows the plasmid map of pgMAX (prokaryotic mode). The pgMAX plasmid was based on pIRESpuro3 (Clontech). The pgMAX plasmid has two functional components, the prokaryotic component for prokaryotic gene expression (lac promoter and lac operator) and for efficient subcloning (Fig 1 prokaryotic unit) and the mammalian expression component, which is composed of the CMV promoter (mammalian unit I) and the internal ribosomal entry site (IRES) puromycin N-acetyl-transferase (Pac) with a polyA tail (mammalian unit II). A lac promoter and lac operator containing SwaI and PmeI restriction sites are under the control of the CMV promoter. At the PmeI site, a Kozak sequence followed by a Flag protein-coding sequence was inserted. A blunt-end DNA fragment can be inserted into the EcoRV blunt-end site within the multiple cloning site, which also contains an inhibitory unit (iUnit).

### Simple subcloning and expression in prokaryotic mode

**Fig 2:**
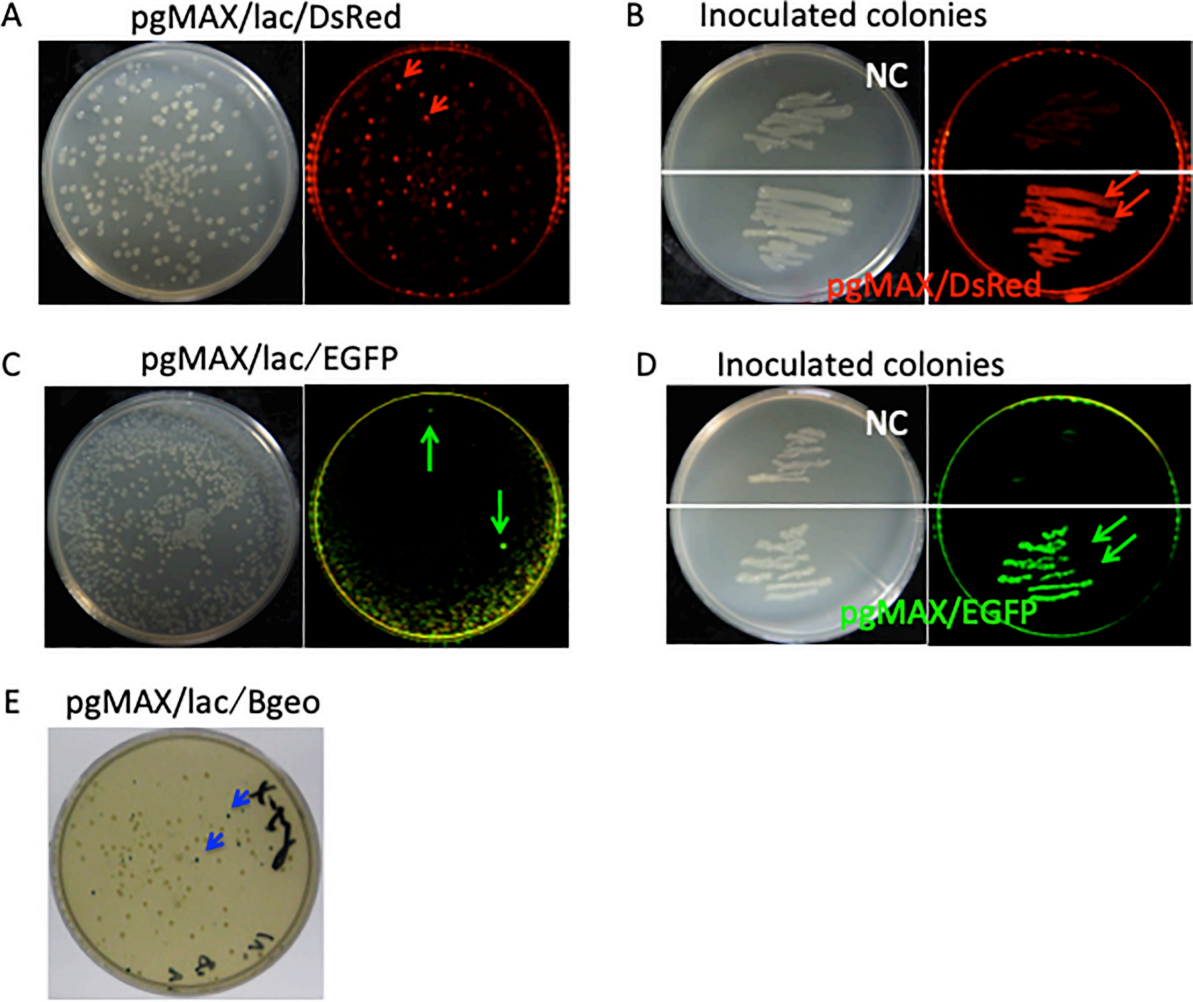
**A. Insertion of the DsRed2 fragment.** Recombinant clones of the pgMAX/blunt-end DsRed2 fragment were transformed into *E*. *coli* on LB plates supplemented with ampicillin and IPTG (left panels). Colonies observed under a white light (left panel) and DsRed2 fluorescence under a green light and a red filter (right panel) are shown. Colonies with red fluorescence are indicated (red arrows). **B. Re-plated DsRed2 containing clone.** Under a green light and a red filter, DsRed2 fluorescence was observed (double red arrows), while the negative control (pgMAX without the DsRed2 fragment) showed no red fluorescence (NC). **C. Insertion of the EGFP fragment.** Recombinant clones of the pgMAX/blunt-end EGFP fragment were transformed into *E*. *coli* on LB plates supplemented with ampicillin and IPTG. Colonies observed under a white light (left panel) and EGFP fluorescence observed under a blue light and orange filter (right panel) are shown. Colonies with EGFP fluorescence are indicated (green arrows). **D. Re-plated EGFP containing clone.** Under a blue light and orange filter, EGFP fluorescence was observed (double green arrows), whereas the negative control (pgMAX without the EGFP fragment) showed no green fluorescence (NC). **E. Insertion of the Bgeo product.** Recombinant clones of pgMAX containing a blunt-end Bgeo fragment were plated onto LB plates supplemented with ampicillin, IPTG and Xgal. Blue colonies are indicated (blue arrows).

First, we examined ligation of the PCR-amplified DsRed2 fragment (ca. 700 bp) into the EcoRV site of pgMAX. After 16 h of incubation on LB agar plates containing ampicillin (150 μg/ml) and IPTG (1 mM for lac operon induction), colonies were observed under green light (excitation wavelength 563 nm) through a filter set (emission wavelength 582 nm). Some colonies showed bright DsRed2-origin fluorescence (Fig 2A, red arrows). Colonies with red fluorescence were inoculated onto LB plates supplemented with ampicillin and IPTG for 16 h. Inoculated colonies containing the DsRed2 insert in the sense direction following the lac promoter exhibited bright red fluorescence (Fig 2B, double red arrows, pgMAX/DsRed).

As a next step, ligation of the PCR-amplified EGFP fragment (ca. 700 bp) was examined (Fig 2C, green arrows). EGFP inserted pgMAX exhibited green fluorescence under blue light (excitation wavelength 470 nm) through a filter set (emission wavelength 505 nm). Because of the background fluorescence of *E*. *coli*, EGFP containing colonies were not as apparent as the DsRed2 containing colonies. Inoculated EGFP colonies exhibited bright green fluorescence (Fig 2D, double green arrows, pgMAX/EGFP).

As DsRed2 and EGFP are relatively short genes, we next examined Bgeo, which is a combination of β-galactosidase and the neomycin phosphotransferase gene (3.0 kb length). A blunt-end Bgeo fragment was inserted into the EcoRV site of pgMAX. The constructed pgMAX/Bgeo showed obvious blue colonies on a plate containing ampicillin, IPTG and Xgal (0.004 mg/ml), indicating the expression of β-galactosidase (Fig 2E blue arrows).

Among all of the 44 randomly picked colonies on the LB agar plates containing ampicillin and IPTG, 42 colonies contained the insert (DsRed2: 8 of 8, EGFP: 12 of 12, Bgeo: 20 of 22 colonies) with an approximately 90% success rate (S2 Table). Using fluorescence (DsRed2 or EGFP) or blue-white (X-gal) selection, all colonies (DsRed2: 8 of 8, EGFP: 8 of 8, Bgeo: 8 of 8 colonies) contained the expected inserts with the desired sense-direction of the lac promoter. Taken together, our data demonstrate that pgMAX is a simple and universal cloning plasmid system for subcloning and prokaryotic (*E*. *coli*) gene expression.

### Simple preparation and expression for mammalian mode

To analyze mammalian expression using the pgMAX system, the pgMAX plasmid with the DsRed2 gene was evaluated. Colonies with red fluorescence were selected and grown in LB medium supplemented with ampicillin (150 μg/ml) for 16 h, and plasmid DNA was purified using standard techniques. Insertion of the DsRed2 fragment was confirmed with restriction enzymes (EcoRI and XhoI) and PCR with specific primers (pgMAXfor and DsRed2rev). The purified plasmid DNAs of these red colonies were further restricted with SwaI and PmeI and re-ligated to delete the lac promoter unit (SwaI-lac promoter-lac operator-PmeI sequence), before being transformed with standard competent cells and cultured for 16 h at 37°C. The presence of the plasmid insert and the deletion of the lac promoter unit (combined sequence of the lac promoter and operator) were confirmed by PCR with specific primers (S1A Fig, lane M; pgMAXfor and DsRed2rev). Plasmid DNA (mammalian mode) was further purified and used for transient expression in HEK293T cells.

**Fig 3:**
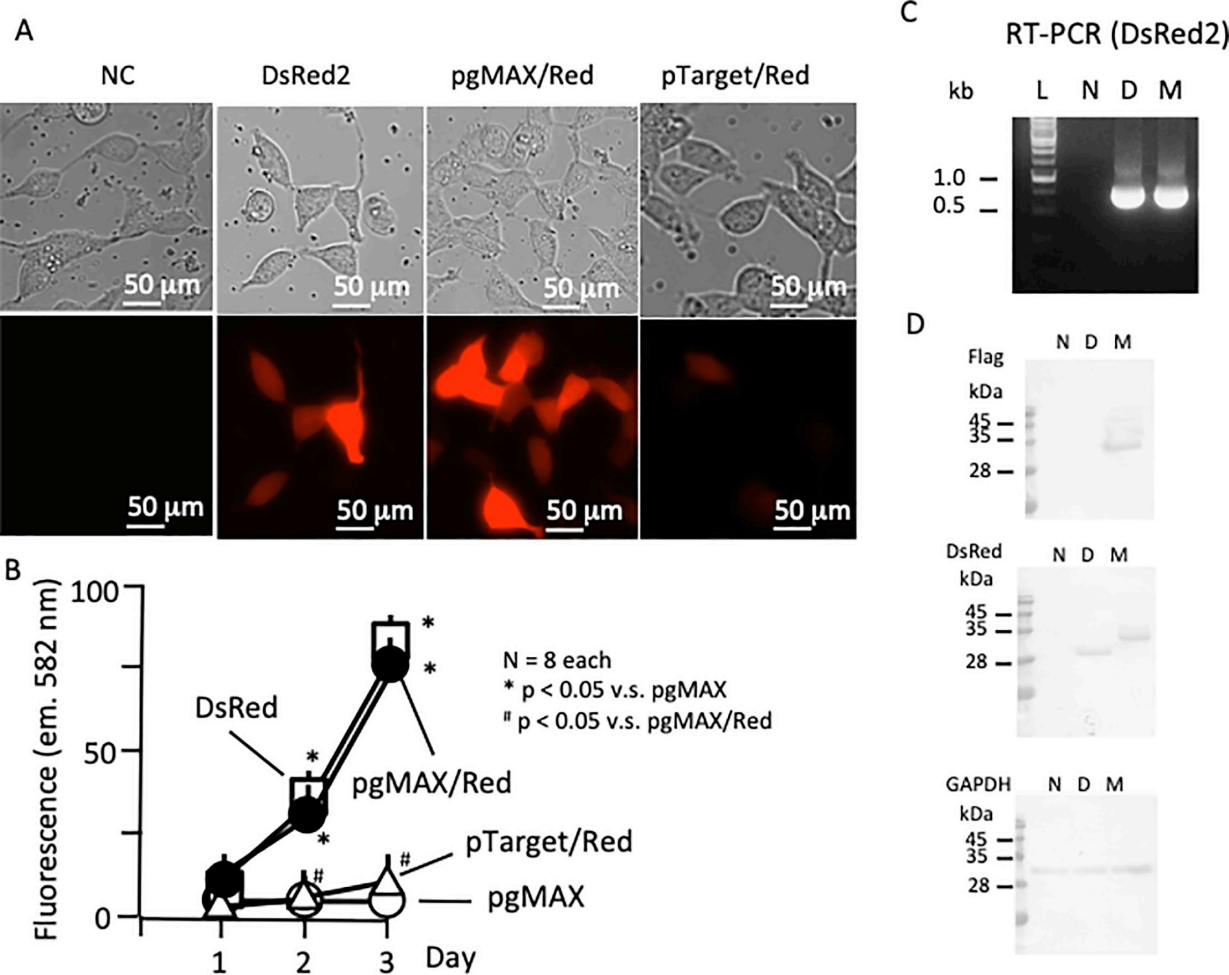
**A. Transfection of pgMAX/DsRed2 (mammalian mode)**. Transfected plasmids are indicated. Phase-contrast (upper panels) and DsRed2 fluorescence (lower panels). pgMAX/DsRed exhibited strong red fluorescence comparable with DsRed2. pTarget/DsRed showed limited red fluorescence. **B. Time-dependent changes in red fluorescence.** Statistical analysis of red fluorescence of DsRed2-transfected cells (open box), pgMAX/DsRed-transfected cells (filled circle) (n = 8), pTarget/DsRed-transfected cells (open triangle) (n = 8), and pgMAX-transfected cells (negative control; open circle) (n = 8). Fluorescence of DsRed2 and pgMAX/DsRed increased up to the third day (3 days after transfection) (*p < 0.05 vs. negative control, ^#^p < 0.05 vs. pTarget/DsRed, n = 8 each construct). **C. Comparative RT-PCR analysis of DsRed2.** Comparative RT-PCR with DsRed2 specific primers resulted in comparable expression of DsRed2 with DsRed2 (D) and pgMAX/DsRed (M). A negative control (N), HEK293T cells (non-transfected) and DNA ladder (L) are shown. **D. Western blot analysis using anti-flag, anti-DsRed and anti-GAPDH antibodies.** The anti-flag antibody recognized a 33-kDa band in pgMAX/DsRed-transfected cells (M). The anti-DsRed antibody recognized a 30-kDa band in DsRed2-transfected cells (D) and a 33-kDa band in pgMAX/DsRed-transfected cells (M). The negative control (N), HEK293T cells (non-transfected), is shown. GAPDH was used as an endogenous control.

After 24 h of plasmid DNA transfection in HEK293T cells, DsRed2-related fluorescence was readily observed with a fluorescence microscope (excitation wavelength 563 nm, emission wavelength 582 nm). The pgMAX plasmids without DsRed2 fragments (negative control) did not exhibit DsRed2 fluorescence (5.1 ± 0.03 units [U], n = 8, Fig 3B, negative control [NC]), whereas red fluorescence was observed when the DsRed2 fragment was present (19.7 ± 1.1 U, n = 8, Fig 3A, pgMAX/Red). As a positive control, a DsRed2 plasmid was also transfected, resulting in comparable fluorescence (17.8 ± 1.7 U, n = 8, DsRed2). The transfection efficiency of pgMAX/DsRed was comparable to that of DsRed2. The pgMAX/DsRed transfected HEK293T cells showed a fair transfection rate (16.1%; 23 of 142 cells, 48 h after transfection), compared to DsRed2 transfected HEK293T cells (9.9%; 15 of 151 cells, 48 h after transfection). As another control, the commercially available pTarget plasmid (Promega) was also used for expression analysis. The PCR-amplified DsRed2 fragment was inserted into the EcoRV site of pTarget with blue-white selection. pTarget with the DsRed2 gene exhibited limited visible red fluorescence compared to DsRed2 or pgMAX/Red (4.9 ± 0.1 U, n = 8, Fig 3A, pTarget/Red), with a lower transfection efficiency (4.2%; 7 of 164 cells, 48 h after transfection).

DsRed2-related fluorescence was further measured with the Orca system, confirming comparable fluorescence intensity with DsRed2 plasmid transfection from day 1 to day 3 (87.4 ± 0.8 and 82.2 ± 1.4 U, DsRed2 and pgMAX/DsRed, respectively, n = 8, p < 0.05 vs. negative control, Fig 2B), while limited fluorescence was observed with pTarget containing DsRed2 (15.9 ± 3.5 U, n = 8, p < 0.05 vs. DsRed2 or pgMAX/Red). The overall fluorescence intensity data are available in S3 Table). Additionally, expression of the DsRed2 gene was evaluated by comparative RT-PCR (Fig 3C). DsRed2 (lane D) and pgMAX/Red (lane M) which showed comparable expression of the DsRed2 gene in HEK293T cells. To examine the universality of pgMAX/Red expression, transient expression of pgMAX/Red was examined in PC3, DU145 and KK47 cells. PC3 and DU145 are prostate cancer cell lines, while KK47 is a bladder cancer cell line. In all three of these cancer cell lines, pgMAX/Red exhibited fairly detectable red fluorescence (S1 Fig). Furthermore, the expression of Flag-tagged DsRed2 was confirmed with anti-Flag and anti-DsRed antibodies (Fig 3D) (the densitometric analysis results are shown in S4 Table). Additionally, puromycin (2 μM) selection was applied after transfection (S2 Fig). In negative control HEK293T cells (without pgMAX plasmid transfection), 24-h puromycin selection resulted in the death of over 90% of the cells, whereas most of the DsRed2-expressing cells survived (S2 Fig). Taken together, the pgMAX plasmid resulted in highly efficient subcloning with prokaryotic expression, easy construction of mammalian expression plasmid vector, and highly efficient selection using the IRES-puromycin resistance gene in cells with the desired gene expression [12].

Additionally, we examined prokaryotic expression of the rabbit voltage-dependent calcium channel β2a subunit using the pgMAX plasmid (S3 Fig). The rabbit β2a subunit cDNA was divided into four fragments (domains) and subcloned into the EcoRV site of pgMAX (S5 Table). The full-length cDNA of the β2a subunit was also subcloned into the same site. The construct was readily transformed into *E*. *coli* and selected using ampicillin and IPTG. Western blot analysis was performed using a commercially available polyclonal antibody specific for the Ca^2+^ channel β2 subunit, which recognized domain D, indicating that the antibody recognized the C-terminal domain of the gene, and that the pgMAX system can be widely adapted (data for the densitometric analysis are shown in S6 Table).

## Discussion

In the present study, we established a new subcloning/expression plasmid (pgMAX-system). This plasmid enables simple and highly efficient subcloning of a desired gene with standard techniques in *E*. *coli* (insertion step and prokaryotic mode), and easy construction of a mammalian expression plasmid within a few days (deletion step: restriction with SwaI and PmeI and re-ligation; after the deletion step, the plasmid is in mammalian mode). The pgMAX plasmid contains several 8-nucleotide rare-cutter enzyme sites that are useful for achieving mammalian mode. In our analysis, the DsRed2-originating PCR fragment exhibited bright red fluorescence, indicating the establishment of a new, simple and efficient expression plasmid system.

The pgMAX plasmid may have several disadvantages. It has a relatively large size (6125 bp). Empirically, the insertion of a DNA fragment into a large plasmid is difficult. Therefore, it may be possible to experience difficulty with the first subcloning step (prokaryotic mode), especially when the insert is large. Furthermore, it is often difficult to produce a sufficient amount of DNA with a large plasmid. It is also important to note that pgMAX requires in-frame ligation into the EcoRV site. In addition, if the desired gene displays toxicity in *E*. *coli*, it might be possible to obtain no colonies with the desired insert. In spite of these disadvantages, we must emphasize the overwhelming advantages of the pgMAX system, including highly efficient subcloning, protein expression in *E*. *coli*, simple construction of mammalian expression plasmids, and efficient and rapid (within 24 h) selection with puromycin. As expressed proteins in *E*. *coli* could be used for immunoblot analysis [13], this plasmid system may be useful for library construction for expression cloning. Puromycin could also be used to establish stable expression cell lines. We also examined direct subcloning of PCR products (without gel purification), such as the TA cloning system, with no success (S2 Table).

To our knowledge, the pTargetT mammalian expression vector (Promega) uses a similar concept to a prokaryotic promoter for efficient blue-white selection and a CMV-related promoter for mammalian expression, although the prokaryotic promoter is inserted in the anti-sense direction. The pTargetT vector functions as both a subcloning and a mammalian expression plasmid. It does not require a deletion step. In our hands, the pTargetT system resulted in simple ligation and prokaryotic expression was not detectable because of the anti-sense direction of the lac promoter, and the level of eukaryotic expression was limited (Fig 3A, pTarget/Red).

On the other hand, pgMAX has a high ligation success rate (over 90% in our experience, S2 Table). It should be noted that expression of the desired gene product was detectable at the prokaryotic level (Fig 2A, 2B and 2C). It is also important to note that the pgMAX system uses only traditional molecular biology apparatuses and reagents. It does not require special substances, which TOPO cloning does [4]. In addition, the pgMAX system could be made to be even shorter (4454 bp) if the IRES-puromycin resistant gene (1661 bp) is deleted.

In the present study, we used an iUnit originating from CcdB, a toxin targeting the essential DNA gyrase of *E*. *coli* [14]. As iUnit selection is quite effective for fragment ligation, pgMAX has significant superiority over classical DNA expression systems with its prokaryotic and mammalian expression modes, as the expressed protein can be examined after the fragment ligation. For simple plasmid construction, we inserted rare restriction enzyme sites (mainly 8-nucleotide rare-cutter enzymes) at both ends of a functional gene, which we call a “unit.” This idea could be widely useful for other types of plasmid construction.

Since the discovery of restriction enzymes and DNA ligase, a number of plasmids have been established [15, 16]. Considering the use of restriction enzymes, the principal idea for the pgMAX plasmid is not novel. Despite this similarity, as we mainly inserted 8-nucleotide rare-cutter enzyme sites (which should be cut every 4^8^ bp) at each end of a gene (lac promoter and operator, iUnit, IRES-puromycin resistance gene), each unit can be easily handled. Taken together, our results indicate that the pgMAX plasmid system enables the simple and easy expression analysis of genes due to its rare-cutter sites.

## Conclusion

We established a new subcloning and expression plasmid with two different modes (prokaryotic and mammalian modes). The new plasmid system enables highly efficient subcloning of a blunt-end DNA fragment, simple expression in *E*. *coli* and that it has a simple deletion step for mammalian expression plasmid construction.

## Supporting information

S1 Table Oligo DNAs used in the construction of pgMAX are shown. (TIFF)

S2 Table Success rate of pgMAX subcloning (cloning of DsRed2, EGFP and Bgeo). (TIFF)

S3 Table Fluorescence intensity values corresponding to Fig 3B. (TIFF)

S4 Table Densitometric analysis of the data presented in Fig 3D. (TIFF)

S5 Table Oligonucleotide sequences used for rabbit voltage-dependent calcium channel β2 subunit expression. (TIFF)

S6 Table Densitometric analysis of the data presented in Supporting Information Fig 3D. (TIFF)

S1 Fig A) Deletion of the lac promoter unit (transfer to mammalian expression mode).
B) Transfection of pgMAX/DsRed in eukaryotic cell lines.
(TIFF)

S2 Fig Transient expression of pMAX/DsRed and puromycin selection in HEK293T cells. (TIFF)

S3 Fig A. Expression analysis of a rabbit voltage-dependent calcium channel β2a subunit in *E. coli*.
B. Western analysis using an anti-voltage-dependent calcium channel β2 antibody.
i) Immunodetection of the interactive domain of the anti-calcium channel β2 antibody.
ii) Coomassie Brilliant Blue(CBB) staining of the total proteins on the gel.
(TIFF)
