# Supplementary figures and images for: A simple and dual expression plasmid system in prokaryotic (E. coli) and mammalian cells
Source: PLoS One. 2019 May 2;14(5):e0216169. doi: 10.1371/journal.pone.0216169 (PMC6497378; doi:10.1371/journal.pone.0216169)

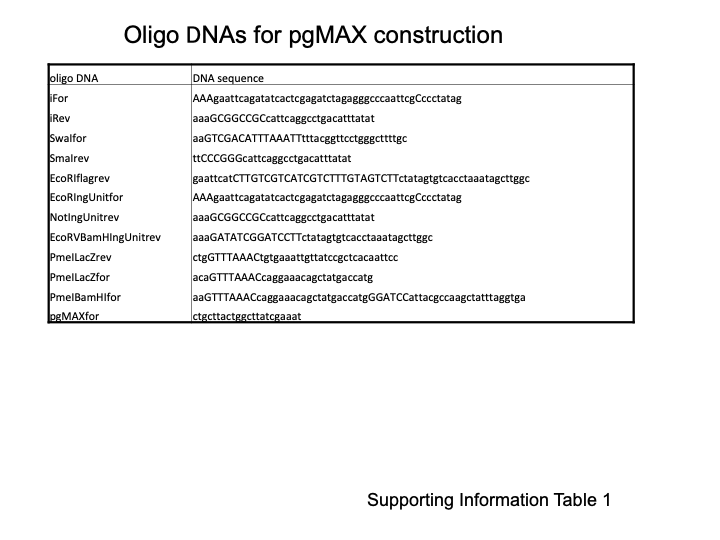

Supplement: S1 Table — (TIFF) [file pone.0216169.s001.tiff]

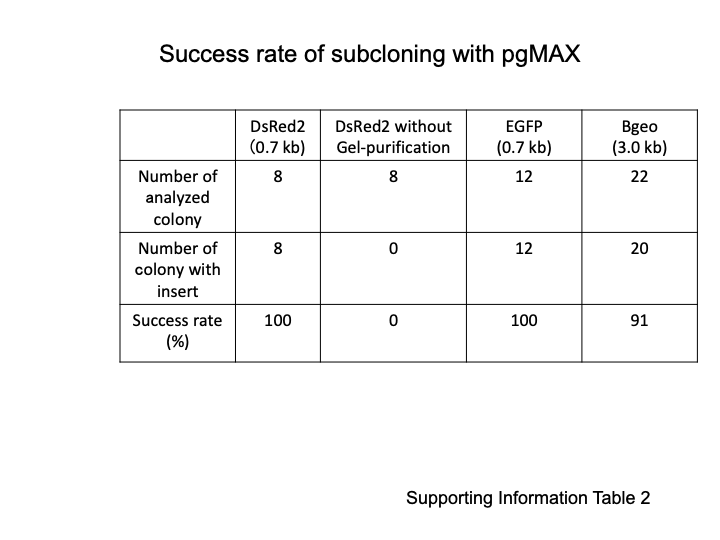

Supplement: S2 Table — (TIFF) [file pone.0216169.s002.tiff]

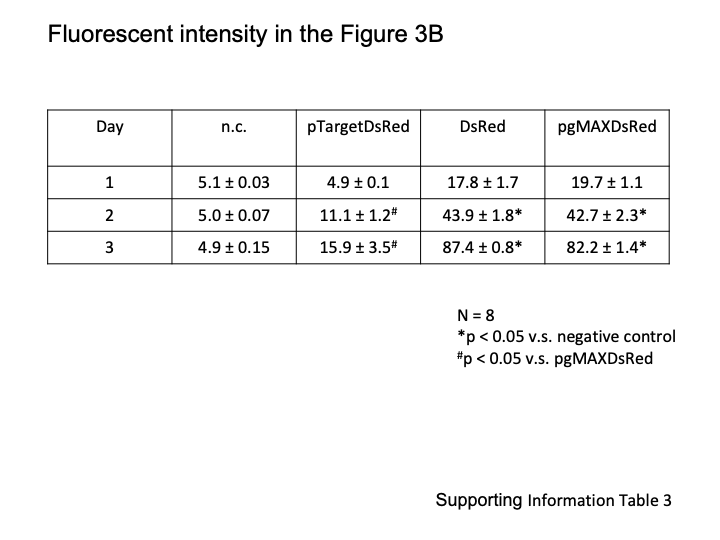

Supplement: S3 Table — (TIFF) [file pone.0216169.s003.tiff]

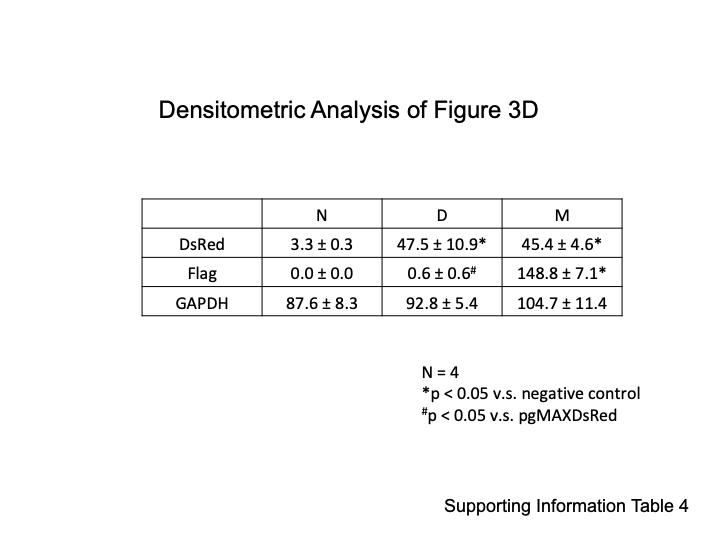

Supplement: S4 Table — (TIFF) [file pone.0216169.s004.tiff]

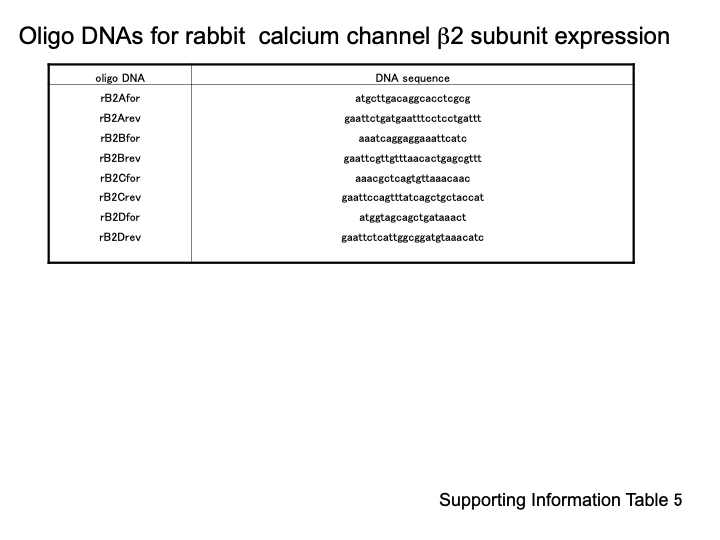

Supplement: S5 Table — (TIFF) [file pone.0216169.s005.tiff]

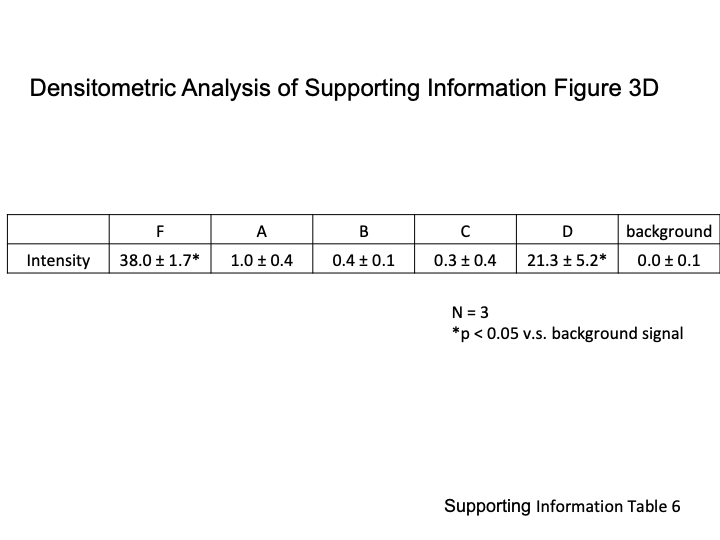

Supplement: S6 Table — (TIFF) [file pone.0216169.s006.tiff]

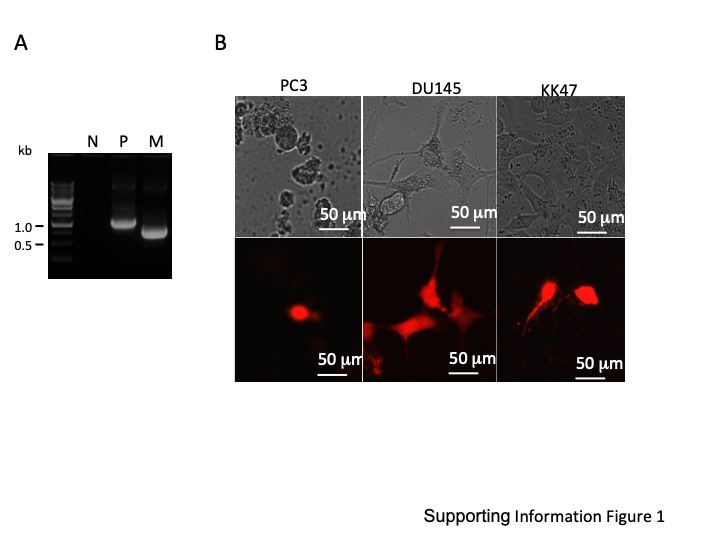

Supplement: S1 Fig — A) Deletion of the lac promoter unit (transfer to mammalian expression mode). B) Transfection of pgMAX/DsRed in eukaryotic cell lines. (TIFF) [file pone.0216169.s007.tiff]

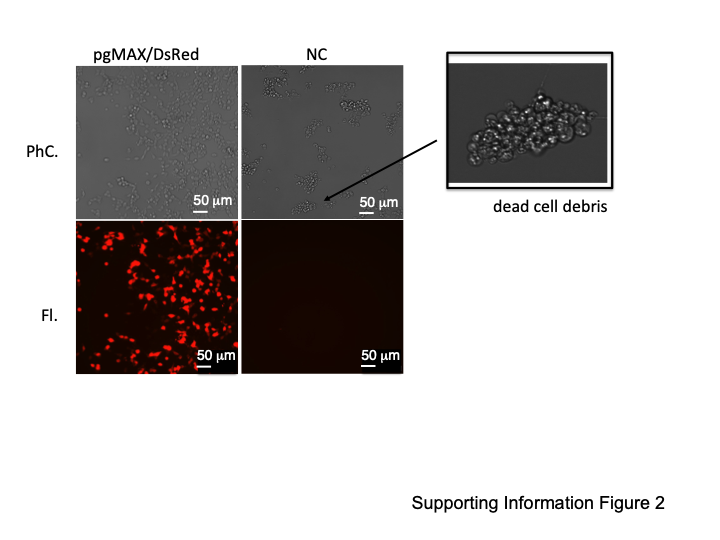

Supplement: S2 Fig — (TIFF) [file pone.0216169.s008.tiff]

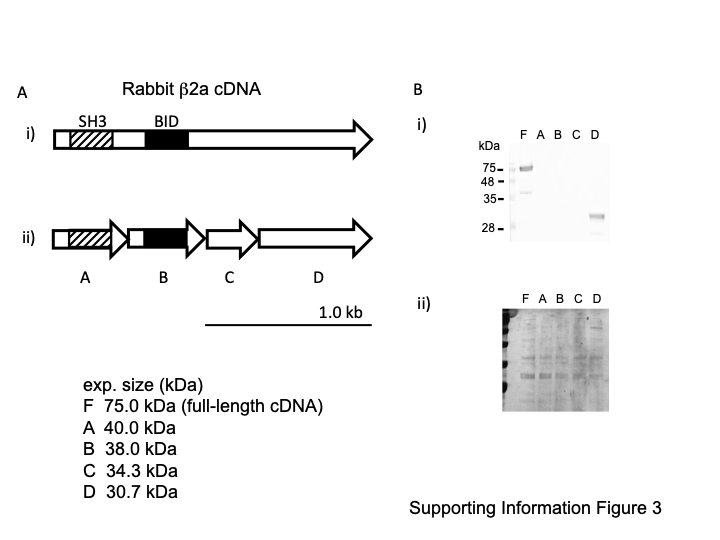

Supplement: S3 Fig — A. Expression analysis of a rabbit voltage-dependent calcium channel β2a subunit in E. coli. B. Western analysis using an anti-voltage-dependent calcium channel β2 antibody. i) Immunodetection of the interactive domain of the anti-calcium channel β2 antibody. ii) Coomassie Brilliant Blue(CBB) staining of the total proteins on the gel. (TIFF) [file pone.0216169.s009.tiff]
